# First real-life data on COVID-19 vaccine effectiveness against hospitalisation and severe disease from the eastern part of the WHO European Region

**DOI:** 10.1016/j.lanepe.2024.101130

**Published:** 2024-11-05

**Authors:** Josip Begovac

**Affiliations:** University Hospital for Infectious Diseases Dr. Fran Mihaljević, Zagreb, Croatia

While randomised trials led to licensing of different COVID-19 vaccines, it is also vital to study vaccine effectiveness (VE) in real-word settings. Those studies can provide robust and important data for the public and shape COVID-19 vaccine policy. The European Centre for Disease Prevention and Control regularly publishes updates on VE from European Union (EU) countries.^1^ However, data on VE from non-EU countries of Western Balkan and Eastern Europe have so far not been reported. There are also differences in COVID-19 vaccine uptake across Europe.

Among EU member countries, vaccine refusals and hesitancy are highest among former communist countries of Central and Eastern Europe.^2^ Yet, vaccine uptake among non-EU member countries of the WHO European region is even lower (Table 1). There are many factors that contribute to vaccine hesitancy. It has been suggested that the lack of trust towards health care professionals and governments plays a major role in post-communist Eastern and Central European countries.^2^

**Table 1:** Vaccination data reported to the World Health Organization by selected European countries.

Country	Latest report date	Population (millions)	Uptake with at least one dose (%)	Uptake with a complete vaccine series (%)	Uptake with first booster dose (%)	Uptake with second booster dose (%)
Selected countries of the WHO European region						
Serbia	05 Mar 2023	6.72	46.6	45.6	0	0
North Macedonia	07 May 2023	1.82	40.6	39.9	7.7	0.4
Kosovo^a^	15 Jan 2023	1.59	50.8	46.3	5.9	0.1
Georgia	28 May 2023	3.81	34.6	31.6	6.7	0
Albania	10 Sep 2023	2.79	47.3	44.8	14.1	2
Kyrgyzstan	18 Jun 2023	7.22	23.4	20.5	4.6	0
Selected Central and Eastern European Union countries						
Slovakia	03 Sep 2023	5.50	52.3	51.4	31	1.4
Czech Republic	03 Sep 2023	10.70	66.3	65.5	41.5	7.6
Poland	31 Dec 2023	38.41	61	60.3	34.3	7.7
Estonia	01 Oct 2023	1.36	63.5	63.2	36.6	8.2
Latvia	13 Aug 2023	1.87	71.7	69.6	29.4	3.6
Lithuania	01 Oct 2023	2.85	69.9	68	32.1	1.3
Hungary	31 Dec 2023	9.66	65.6	63.5	40	4.3
Romania	03 Dec 2023	18.98	43	42.8	9.3	0.2
Croatia	01 Oct 2023	3.89	60.1	58.2	25.8	1.9
Slovenia	31 Dec 2023	2.12	57.8	56.3	32.3	3.7
Bulgaria	26 Nov 2023	6.74	31.5	30.4	12.6	2

a Reference to Kosovo should be understood to be in the context of the United Nations Security Council resolution 1244 (1999).

In this issue of *The Lancet Regional Health – Europe* Katz MA et al.^3^ report data on VE against hospitalisation from 5126 hospitalised adults meeting the WHO severe acute respiratory infection (SARI) case definition^4^ from 25 hospitals from six middle-income countries of the eastern WHO European region (Albania, Georgia, North Macedonia, Serbia, Kosovo, and Kyrgyzstan). The study was conducted between 1st January 2022 and 23rd November 2023, at a time when many people might have acquired some immunity because of past SARS-CoV-2 infection. In their analytical approach, the authors followed the WHO recommendations on how to conduct a VE test-negative study.^4^ A test-negative case–control study implies that persons hospitalised for SARI are compared based on whether SARS-CoV-2 was detected by a molecular test. Those who test positive are cases and those who test negative are controls. VE against hospitalisation is then calculated by comparing the odds of vaccination between cases and controls, while adjusting for confounders. The test-negative design also enables estimation of the duration of VE. In this study, a total of 1009 persons tested positive for SARS-CoV-2 and the VE against hospitalisation was 60.1% (95% confidence interval: 12.4–81.8) for a last dose received in the preceding 3 months. Severe disease, which included individuals who required oxygen, those on mechanical ventilation, those admitted to intensive care, and those who died in the hospital, was also halved (53.9%, 95% confidence interval: 26.9–70.9) if the last vaccine dose was received in the preceding 14–179 days. The reported overall VE was a result of an analysis of six different COVID-19 vaccines, some of which have not been approved for use in the EU. The use of such vaccines has also been reported in a survey of 22 countries of Central and Eastern Europe.^5^ As expected, the VE waned over time and was essentially absent after 6 months. “Annual” VE (ie, VE of any COVID-19 vaccine received in the past 12 months) was similar to the more often reported absolute and relative VE estimates, supporting the WHO recommendation for revaccination 6–12 months after the most recent dose.^6^

The study has limitations which are extensively acknowledged and include a relatively small number of participants, implying that the study was underpowered to assess VE for each individual vaccine or report robust VE separately in the elderly or across different Omicron subvariants.

SARS-CoV-2 is constantly evolving. Currently, the dominant subvariant of interest in Europe is from the KP.3 Omicron lineage.^7^ However, despite the constant evolution of the virus and the fact that strains used in the vaccine often do not match the circulating strain, protection against severe disease is still present. This is explained by the presence of memory B and T cells, as T-cell epitopes are largely conserved across variants.^8^

There is also emerging data showing that the incidence of hospitalisation and death is higher in reinfected unvaccinated persons compared to those vaccinated with a breakthrough infection, suggesting that vaccine-induced immunity provides better protection than naturally acquired immunity.^9^ Risk factors for severe COVID-19 have not changed since the beginning of the epidemic and older age and comorbidities drive hospitalisations, critical disease, and deaths.^10^ We live in an era of vaccine hesitancy and it seems that the public health message about vaccine effectiveness is almost disappearing. This is why local data, albeit confirming findings from other regions in the world, are important. This study provides an additional argument for voicing a stronger public health message about the importance of COVID-19 vaccination in the eastern part of the WHO European Region.

## Declaration of interests

None.
